# Field trial to correlate mineral solubilization activity of *Pseudomonas aeruginosa* and biochemical content of groundnut plants

**DOI:** 10.1515/biol-2022-1008

**Published:** 2025-04-22

**Authors:** Sunitha Kumari Krishnan Kutty, Padma Devi Skandasamy Natchimuthu, Rajamani Ranjithkumar, Sinouvassane Djearamane, Lai-Hock Tey, Ling Shing Wong, Saminathan Kayarohanam, Natarajan Arumugam, Abdulrahman I. Almansour, Sakkarapalayam M. Mahalingam

**Affiliations:** Department of Botany, PSGR Krishnammal College for Women, Peelamedu, Coimbatore, 641 004, Tamil Nadu, India; Department of Pharmacology, Saveetha Medical College and Hospital, Saveetha Institute of Medical and Technical Sciences (SIMATS), Saveetha University, Chennai, 602105, Tamil Nadu, India; Faculty of Science, Universiti Tunku Abdul Rahman, Jalan Universiti, Bandar Barat, Kampar, 31900, Malaysia; Biomedical Research Unit and Lab Animal Research Centre, Saveetha Dental College, Saveetha Institute of Medical and Technical Sciences, Saveetha University, Chennai, 602 105, India; Faculty of Health and Life Sciences, INTI International University, Persiaran Perdana BBN, Putra Nilai, 71800 Nilai, Negeri Sembilan, Malaysia; Faculty of Bioeconomics and Health sciences, Geomatika University Malaysia, Kuala Lumpur, 54200, Malaysia; Department of Chemistry, College of Science, King Saud University, P.O. Box 2455, Riyadh 11451, Saudi Arabia; Department of Chemistry, 720 Clinic Drive, Purdue University, West Lafayette, Indiana, 47907, United States of America

**Keywords:** *Pseudomonas aeruginosa*, zinc, phosphorus, solubilization, field study, carbohydrates, proteins, chlorophyll, oil, soil, fertility

## Abstract

The excessive use of phosphorus (P) fertilizers increases crop production but can lead to P-induced zinc (Zn) deficiencies, making both nutrients unavailable to plants. Plant–microbe interactions, such as with *Pseudomonas aeruginosa*, can alleviate these constraints by solubilizing Zn and P in soil. A soil incubation study revealed that applying *P. aeruginosa* with farmyard manure (FYM) significantly increased Zn and P solubilization (6.86 mg/l; 14.83 mg/l) compared to control (3.15 mg/l; 13.67 mg/l). A field experiment evaluated the effects of *P. aeruginosa* on the biochemical composition of groundnut plants under five treatments. The T2, T3, and T4 treatments had the highest protein, carbohydrate, and chlorophyll levels, likely due to the heterogeneous activity of FYM and the mineral solubilizing ability of *P. aeruginosa*. Groundnut seeds from T3 (combined liquid inoculant and FYM) had the highest iodine (88.47 mg KOH/g), saponification value (195.56 mg KOH/g), and free fatty acid content (2.23 g oleic acid). The pH of the T3 soil decreased from 8.3 to 7.5, and significant increases were observed in electrical conductivity (from 2.88 to 0.30 dS/m), calcium carbonate (2.53–1.7%), organic carbon (0.39–1.91%), nitrogen (273.75–788.25 kg/ha), P (20.1–59.65 kg/ha), potassium (182.25–346.5 kg/ha), and Zn (1.53–7.24 mg/kg). The study suggests that the combined application of liquid formulants of *P. aeruginosa* with FYM is advantageous, as FYM supports microbial growth by providing essential nutrients for mineralization. Moreover, liquid inoculants formulated with polyvinylpyrrolidone as an osmo protectant demonstrated enhanced shelf-life and mineral solubilization, contributing to improved biochemical properties in groundnut plants.

## 1 Introduction

The presence of fourteen mineral elements in the soil is essential for plant growth as they regulate plant metabolic processes and produce crops with excellent quality and yield. They can be categorized into macronutrients like nitrogen (N), phosphorus (P), potassium (K), calcium (Ca), magnesium (Mg), and sulfur (S) and micronutrients like chlorine (Cl), boron (B), iron (Fe), manganese (Mn), copper (Cu), zinc (Zn), nickel (Ni), and molybdenum (Mo) [1]. Farmers focus more on macronutrients since they are critical for plant development and growth, ranging from structural elements to redox-sensing agents [2]. Despite being crucial for crop quality and productivity, providing inherent protection against disease and adverse conditions, micronutrients have received less attention. They also act as co-factors for several enzymes involved in metabolizing various organic compounds, including proteins, lipids, carbohydrates, and nucleic acids [3,4,5]. Micronutrient deficiencies in the soil are a universal phenomenon these days due to the crop’s inability to absorb micronutrients, as well as positive or negative interactions with macronutrients accumulating in the soil as a result of excessive application of chemical fertilizers by the agrarian communities [6,7,8,9]. It is estimated that 49, 31, 15, 14, 10, and 3% of agricultural soils worldwide are deficient in Zn, B, Mo, Cu, Mn, and Fe, respectively [10,11]. Of the micronutrients, only Zn is directly linked in the food chain such that deficiency is extensive in humans, food crops, and soil. The Zn deficiency is therefore the highest priority among micronutrients for agriculture to address [12].

Plants require Zn as a micronutrient because it is vital to many of their bio-physicochemical processes, including protein synthesis, gene regulation and activation, carbohydrate metabolisms, morphological and anatomical participation in bio-membranes, and numerous other critical cellular processes like ion homeostasis, metabolic and physiological processes, and enzyme activation [13,14,15]. Zn must therefore be properly absorbed, transported, and distributed throughout plant tissues, cells, and intracellular spaces to sustain optimal growth and development [16]. Stunted growth and development, chlorosis, necrosis, membrane breakdown, poor metabolism, nutritional imbalance, and increased vulnerability to biotic and abiotic stresses are the symptoms of Zn deficiency in plants [17]. Some of the factors that affect the quantity of Zn available to plants are soil type, soil pH, and the presence of other minerals that compete with Zn absorption, one such interaction is between Zn and P [18]. The Zn-induced P deficiencies are uncommon because farmers usually use more P fertilizer than Zn, which could prevent Zn from being absorbed [19]. According to Sharma et al. [20], precipitation on Fe, Al, and Ca complexes causes 75–90% of the applied P fertilizer to accumulate in the soil. The formation of H^+^ ions by phosphate salts causes the soil’s phosphate content to rise, which in turn prevents Zn from being absorbed by plants due to their co-precipitation as Zn phosphate (Zn_3_(PO_4_)_2_) in both alkaline soils (submerged conditions) and acidic soils. This causes Zn an inaccessible soil element to be absorbed by plants [21,22].

The Zn and P are essential for the metabolism of carbohydrates in plants, to catalyze the enzymes such as carbonic anhydrase, fructose-1,6-bisphosphate, and aldolase. The rapid drop-in activity of these three enzymes slows the metabolism of carbohydrates in plants [21,22,23,24,25]. Nicotinamide adenine dinucleotide and nicotinamide adenine dinucleotide phosphate (NADP), the crucial nutrients involved in plant glucose metabolism, are made up exclusively of P [26]. The ribosome, a location for protein synthesis, disintegrates without Zn because it is part of its structural makeup. Protein synthesis and protein levels are severely reduced in Zn-deficient plants, which results in the accumulation of free amino acids, as Zn is essential for synthesizing numerous groups of amino acids into enzymes and proteins [25,27,28,29]. Consequently, a deficiency in Zn prevents the plant from producing specific enzymes and proteins. P, which is a crucial component of adenosine triphosphate (ATP) and aids in the activation of amino acids for protein synthesis, operates similarly [26].

The availability of mineral nutrients to plants also affects the production of chlorophyll. The dynamics of leaf surface development and area are greatly influenced by mineral nutrition, which is reflected in leaf surface area, photosynthetic capability, and net photosynthetic productivity. Among the minerals necessary for photosynthesis, Zn and P are essential for the production of chlorophyll in plants. Zn is a key enzyme co-factor necessary for the regular functioning of the photosynthetic apparatus and plays a significant role in the control of chlorophyll production in plants. In addition, P contributes to the stability of the chlorophyll molecule as a crucial component [30].

For millions of people around the world, groundnuts (*Arachis hypogaea* L.) are a significant source of cooking oil. As integrated food components [31] and oleo chemicals [32], they give food products a distinct flavor and texture. In seeds, Zn plays a significant role in the production of proteins and lipids [33]. Groundnut-growing soils in India have significant yield loss owing to a 50% Zn shortfall [34,35]. Zn deficiency in groundnuts results in interveinal yellow-vein chlorosis and uneven mottle on upper leaves. The entire leaflet turned chlorotic in conditions of severe deficit [36].

The Zn fertilization boosts nodulation, chlorophyll content, and pod yield in soils with low levels of Zn. According to ILZRO [37], a deficit impaired pegging but had no overt leaf symptoms. Numerous enzymes are activated by Zn [38,39], and this can directly affect developmental processes that result in noticeable variations in seed oil quality. The P is also necessary for the generation of high-quality seeds because it is a coenzyme in reactions that transfer energy; in photosynthesis, energy is used in the form of ATP and NADP. Following that, this energy is utilized in the photosynthetic fixation of CO_2_ and the production of lipids and other vital organic molecules [26].

Thus, employing Zn and P becomes crucial for plant development as they are necessary for controlling the biochemical activities in plants. Even though they accumulate in the soil, they are often found in insoluble forms as a result of their antagonistic interactions. The relationship between the plant and microbes is self-sustaining; the former uses the latter to extract soluble nutrients from the soil, which increases soil fertility. In the rhizosphere, microorganisms that can convert a variety of unavailable metal forms into available ones can be isolated and used to supply plants with Zn and P in their stable forms.

*Pseudomonas aeruginosa* is a rhizobacterium that stimulates plant development by secreting phytohormones, siderophores, hydrogen cyanide, and lytic enzymes, solubilizing P, K, and Zn, and so gaining great attention in the field of Biofertilization [40]. Therefore, in the current investigation, the impact of *P. aeruginosa*’s mineral solubilizing activity on the biochemical content of the groundnut plants cultivated under various treatments at regular 30-day intervals was examined.

## 2 Materials and methods

Laboratory assessment of the Zn-solubilizing ability of *P. aeruginosa* was carried out qualitatively and quantitatively under different cultural conditions [41,42]. Field experiments were also conducted to test its influence on the growth and yield of *A. hypogaea* [43].

The primary goal of the present investigation was to evaluate the impact of *P. aeruginosa*’s mineral (Zn and P)-solubilizing activity on the biochemical characterization of *A. hypogaea* L. A field study was performed on an agricultural farm at Kangeyam, Tirupur District, Tamil Nadu. The *P. aeruginosa* was formulated in a variety of ways, including solid forms employing lignite at a rate of 100 ml (10^9^ CFU/ml) per 200 g and liquid forms using 3% poly vinyl pyrrolidone (PVP), which has a cell load of 10^9^ CFU/ml. Before sowing, 100 seeds were treated with 5 g of solid-based inoculant with a cell load of 10^9^ CFU/ml and also 5 ml of liquid inoculum with a cell load of 10^9^ CFU/ml per 100 seeds and incubated for 6 h at room temperature.

The experimental field was set up using a randomized complete block design with five treatments (T1: control [uninoculated seeds]; T2: seeds treated with liquid inoculant; T3: seeds treated with liquid inoculant and the soil amended with farmyard manure [FYM]; T4: soil amended with FYM alone; T5: seeds treated with lignite [solid] based bioinoculant) replicated four times on a 3 m by 3 m plot size. At regular intervals of 30 days, *P. aeruginosa*’s effects on the biochemical components of the randomly chosen plants were investigated.

### 2.1 The efficiency of *P. aeruginosa* in solubilizing minerals (Zn and P) present in the soil reserve (soil incubation study) [44]

The *P. aeruginosa*’s effects on the release of Zn and P from the soil reserve were investigated through a soil incubation study using field soil that was taken from an agricultural farm in Kangeyam, Tirupur District, Tamil Nadu. A 1 kg of field soil was taken in a series of pots. FYM was applied to one set of pots at a rate of 5%, while another set was left untreated. The test bacterial culture was centrifuged, and the supernatant solution was discarded after the bacteria were cultured in nutrient broth for 48 h. The bacterial pellets were rinsed with sterile distilled water, and 10 ml (10^9^ CFU/ml) of cell suspension per 1 kg of soil was used to inoculate the pots. Additionally, an uninoculated control was also maintained. Throughout the incubation phase, the soil’s moisture content was maintained. Minerals were extracted and their Zn and P availabilities were evaluated after 10 days of incubation.

#### 2.1.1 Estimation of soil’s available Zn [45]

Diethylene triamine penta acetic acid (DTPA) solution (0.005 M) in a 1:2 ratio was poured into 20 ml of a 100 ml Erlenmeyer flask containing 10 g of soil, and the mixture was then agitated for 2 h. The extract was obtained after the contents were filtered via the Whatman No. 42 filter paper. The amount of available Zn in the soil was measured using an atomic absorption spectrometer.

##### 2.1.1.1 Preparation of DTPA extraction solution

To make the DTPA extraction solution, 200 ml of distilled water was used to dissolve 149.2 g of 0.1 M triethanolamine, 19.67 g of 0.005 M DTPA, and 14.7 g of 1 M CaCl_2_·2H_2_O. This solution was then concentrated up to 10 l. 1 N HCl was used to bring the pH down to 7.3 ± 0.05.

#### 2.1.2 Estimation of available P in soil

The available P in soil was estimated by Olsen’s method [46].

##### 2.1.2.1 Reagents preparation

- Sodium bicarbonate (0.5 M),
- Activated carbon, and
- 5 N sulfuric acid.

Conc. H_2_SO_4_, 137 ml was added to 1 l of distilled water

##### 2.1.2.2 Reagent A

(a) Ammonium molybdate (12 g) was dissolved in distilled water (250 ml).
(b) Antimony potassium tartrate (0.291 g) was dissolved in distilled water (100 ml).
(c) 100 ml of 5 N H_2_SO_4_ was prepared by dissolving 137 ml of conc. H_2_SO_4_ in 1 l of distilled water.
(d) The three reagents were mixed as described above and the volume was increased up to 2 l with distilled water.

##### 2.1.2.3 Reagent B

Ascorbic acid (1.056 g) was dissolved in reagent A (200 ml).

##### 2.1.2.4 Procedure

One teaspoon of activated carbon and 50 ml of 0.5 M sodium bicarbonate was added to a 100 ml Erlenmeyer flask containing 5 g of soil. The mixture was filtered through Whatman No. 40 filter paper after being agitated for 30 min in an orbital shaker. To get a clear filtrate, additional activated carbon may have to be added. Using 5 N H_2_SO_4_, 5 ml of filtrate was pipetted into a 25 ml volumetric flask and acidified to pH 5.0. After the mixture was diluted to 20 ml, 4 ml of freshly prepared reagent B was added, and the volume was then increased to 25 ml with distilled water. The flask was then thoroughly shaken and let to stand for 10 min. In a Vis spectrophotometer, the resulting blue color’s absorbance was detected at 660 nm. Distilled water was used as blank. The available P is represented in mg/l, and the unknowns were determined using the standard graph.

### 2.2 Biochemical characterization of the groundnut plants

The biochemical contents of the fresh leaves and seeds of groundnut plants such as total carbohydrate, soluble protein, total chlorophyll content of the leaves, oil percentage, iodine value, saponification value, and free fatty acids content of the oil extracted from the groundnut seeds were assessed at regular 30-day intervals in triplicates.

#### 2.2.1 Total carbohydrate

The total carbohydrate content of groundnut plant leaves and seeds was evaluated by the Hedge and Hofreiter method [47]. A 1 g of the fresh sample (leaves as well as seeds of groundnut plants) was hydrolyzed individually in a water bath for 3 h with 2.5 N HCl (5 ml), and after cooling to room temperature, it was neutralized with Na_2_CO_3_. 4 ml of Anthrone was added to 0.1 ml of individual extracts in test tubes after they had been prepared to a volume of 1 ml with distilled water. Color intensity at 630 nm was measured after the tubes had been kept boiling for 5 min. The glucose standard graph was used to compute the total carbohydrate concentration of the samples.

#### 2.2.2 Soluble protein

By using Bradford’s method [48], the total soluble protein in fresh leaves and seeds was determined. The samples were weighed at 0.1 g, homogenized in 2 ml of 0.1 M phosphate buffer pH 6.8 in a mortar and pestle that had been pre-chilled while homogenization was taking place, maintained on ice, then centrifuged at 5,000 rpm for 10 min at 4°C. Equal parts of 20% trichloroacetic acid that had already been cooled were added to the supernatant before being centrifuged once more at 5,000 rpm. The particle was rinsed with acetone and then dissolved in 1 ml of 0.1 N NaOH after the supernatant was discarded. For optimal color development, tubes were covered with aluminum foil and kept in the dark, and 5 ml of Bradford reagent was added to a 1 ml aliquot. The amount of soluble protein was estimated and represented as mg/g using the standard bovine serum albumin (Sigma-Aldrich) as a reference.

#### 2.2.3 Chlorophyll content of the leaves [49]

##### 2.2.3.1 Extraction of chlorophyll

A 20 ml of 80% acetone was used to grind one gram of fresh leaves. It was then centrifuged for 5 min at 5,000 rpm. The process was repeated until the residue was colorless after the supernatant was transferred. At 645 and 663 nm, the solution’s absorbance was read in comparison to a solvent (acetone) blank.

##### 2.2.3.2 Estimation of chlorophyll content

The following equation was used to determine the levels of chlorophyll *a* (1), chlorophyll *b* (2), and total chlorophyll (3):

(1) \[\text{Chlorophyll}\hspace{.5em}\text{a}:\hspace{.5em}12.7(\text{A}663)\hspace{.5em}\mbox{--}\hspace{.5em}2.69(\text{A}645),]\]

(2) \[\text{Chlorophyll}\hspace{.5em}\text{b}:\hspace{.5em}22.9(\text{A}645)\hspace{.5em}\mbox{--}\hspace{.5em}4.68(\text{A}663),]\]

(3) \[\text{Total}\hspace{.5em}\text{Chlorophyll}:\hspace{.5em}20.2(\text{A}645)\hspace{.5em}+\hspace{.5em}8.02(\text{A}663).]\]

#### 2.2.4 Extraction of groundnut oil

Groundnut seeds were powdered using a laboratory Ball Mill. Using petroleum ether (60°C) for 6 h, oil was extracted using the Soxhlet technique [50].

##### 2.2.4.1 Determination of percentage yield

The percentage (%) yield was calculated using [51]

\[\text{Percentage}\hspace{.25em}\text{yield}\hspace{.25em}\text{of}\hspace{.25em}\text{oil}=\frac{\text{Weight}\hspace{.25em}\text{of}\hspace{.25em}\text{oil}}{\text{Weight}\hspace{.25em}\text{of}\hspace{.25em}\text{sample}(\text{g/nut})}\times 100.]\]

##### 2.2.4.2 Iodine value

According to the AOAC method [51], the iodine value of oils was determined. A conical flask containing 0.25 g of oil was filled with a 10 ml solution of CCl_4_. Similarly, 30 ml of the Hanus solution was added, and the mixture was left to stand for 45 min while being occasionally shaken. Any free iodine on the stopper was rinsed down with 10 ml of 10% KI solution and 100 ml of distilled water. The iodine was titrated using a previously standardized Na_2_S_2_O_3_ solution, which was added gradually while being constantly shaken until the yellow solution became nearly colorless. The addition of a few drops of the starch indicator was followed by a series of titrations until the blue color completely vanished. Iodine that was still present in the CCl_4_ solution was vigorously shaken out of the bottle so that it could be absorbed by the KI solution. The amount of Na_2_S_2_O_3_ solution needed for the experiment was recorded. Along with the sample, a control experiment was conducted.

The following calculation was used to determine the percent weight of iodine that the oil sample had absorbed:

\[I\hspace{.25em}=\frac{(B-A)\times N\times 0.127\times 100}{W},]\]

1 ml 1 N Na_2_S_2_O_3_ = 0.127 g; *B* = ml of 0.1 N Na_2_S_2_O_3_ required by blank; *A* = ml of 0.1 N Na_2_S_2_O_3_ required by oil sample; *N* = normality of Na_2_S_2_O_3_; and *W* = weight of oil in g.

##### 2.2.4.3 Saponification value

To calculate the saponification value, 1.0 g of oil was placed in a conical flask together with 25 ml of 0.5 N alcoholic KOH, which was then heated under a reserved condenser for 30–40 min to ensure that the sample was completely dissolved. Phenolphthalein was added and titrated with 0.2 N HCl after chilling the sample until a pink endpoint was obtained [52].

The saponification value was calculated by the following formula:

\[\text{Saponification}\hspace{.25em}\text{value}\hspace{.25em}(S)=\frac{(B-T)\times N\times 56.1}{W}]\]

*S* = saponification value; *B* = ml of HCl required by blank; *T* = ml of HCl required by oil sample; *N* = normality of HCl; *W* = weight of oil in g; and 56.1 = equivalent weight of KOH.

##### 2.2.4.4 Free fatty acids content of the oil

Around 2 ml of 1% phenolphthalein solution was added to 20 ml of ethanol–diethyl ether (1:1 v/v) mixture, and the mixture was neutralized with 0.10 M NaOH solution. The neutralized mixture was then combined with 5 g of each oil sample, and the combination was titrated against a 0.10 M NaOH solution while being constantly shaken until a pink color appeared and lasted for 15 min. The free fatty acid value was calculated using the titer values [53].

### 2.3 Analysis of the characteristics of soil samples subjected to different treatments

The different parameters of the soil samples subjected to different treatments such as pH, electrical conductivity (EC), CaCO_3_, organic carbon, N, P, K, and Zn were analyzed before and after the cultivation of the groundnut

#### 2.3.1 pH

In a conical flask, 10 g of soil and 100 ml of distilled water were combined, and the mixture was shaken vigorously for 30 min. It was filtered through muslin cloth after 30 min, and the filtrate was used to calculate the pH using an Elico pH meter.

#### 2.3.2 EC

The soil water ratio of 1:10 was used to determine the EC of the soil samples, and the results are expressed in dS/m, according to the method of Jackson [54].

#### 2.3.3 CaCO_3_

The soil samples were crushed and sieved through a 2 mm sieve after being air-dried. As stated by Dreleimanis [55], the equivalent amount of calcium carbonate (E-CaCO_3_) was measured using 0.5 M HCl for dissolving CaCO_3_ and determined between the two titrations of the surplus acid by using 0.2 M NaOH.

#### 2.3.4 Organic carbon

The titration method, recommended by Walkley and Black [56], was used to determine the amount of organic carbon in the soil samples.

#### 2.3.5 N content

The Kjeldahl method was used to determine the soil sample’s total N content, and the calculation was made per Vogel’s [57] recommendations.

#### 2.3.6 K content

By using the titration method recommended by Jackson [54], the K content of the soil sample was calculated.

#### 2.3.7 Zn

By using the method outlined in Section 2.1.1, the available Zn present in the soil samples was determined.

#### 2.3.8 P

By using the method outlined in Section 2.1.2, the available P present in the soil samples was determined.

### 2.4 Statistical analysis

A randomized complete block design with four replicates of each treatment was used to set up plots with various treatments in field trials. Analysis of variance was used to analyze the data, and Duncan’s multiple range test was used to compare means at *p* ≤ 0.05. The SPSS software (2019 version) was used for all analyses.

## 3 Results and discussion

### 3.1 Efficiency of *P. Aeruginosa* in solubilizing minerals (Zn and P) present in the soil reserve (soil incubation study)

This investigation was carried out to ascertain the impact of *P. aeruginosa* inoculation to solubilize Zn and P present in the soil. The results showed no significant difference in the mineral solubilizing ability of bacteria that is applied alone versus applied along with FYM. But the efficacy of the organism to solubilize Zn was little enhanced when it was applied along with FYM (6.86 mg/l) compared to mineral solubilizing bacteria alone (6.02 mg/l) and control (3.15 mg/l) (Table 1). By providing a nutrient-rich environment that supports the growth and activity of this beneficial microbe (*P. aeruginosa*), and by enhancing their effectiveness in producing organic acid, which may have increased Zn availability in the soil, FYM application has the potential to increase available Zn concentrations beyond the deficiency threshold limits. Several forms of Zn, including ZnO, ZnCO_3_, and ZnS, that are present in the soil can be dissolved by the Zn-solubilizing bacterial strains producing organic acids mainly 2-ketogluconic acid and gluconic acid [58,59]. Furthermore, FYM has a significant role in regulating temperature, retaining moisture, increasing soil organic carbon (SOC) availability, and improving nutrient availability and soil pH [60,61,62]. These actions and conditions generate an environment that is conducive to microbial growth and activity [63,64]. Since certain Zn-solubilizing microorganism (ZSM) species are copiotrophic which favor habitats that are high in carbon and nutrients, the addition of FYM may increase the availability of carbon and nutrients in the soil, which will in turn encourage the activity of copiotrophic ZSM species in soil [65,66]. Applying FYM and Zn-solubilizing bacteria raised the soil’s DTPA-Zn content, as reported by Senthil Kumar et al. [67]. ZSMs are therefore viable substitutes for increasing Zn bioavailability and supplementing plants in an economical, environmentally responsible, and sustainable way [68,69].

**Table 1:** Release of minerals in the soil inoculated with *P. aeruginosa*

Treatments	Amount of minerals (mg/l)	
	Zn	P
Control	3.15 ± 0.50^b^	13.67 ± 0.58^b^
MSB	6.02 ± 0.18^ab^	14.33 ± 0.29^ab^
MSB + FYM	6.86 ± 0.03^a^	14.83 ± 0.29^a^
SD CD (*p* < 0.05)	0.2511	0.3333
	0.6145	0.8157

Values are mean ± SD of three samples; MSB: mineral-solubilizing bacteria; FYM: farmyard manure.

Regarding the amount of P that was readily available, inoculating *P. aeruginosa* with FYM led to the highest concentration (14.83 mg/l), surpassing both the control (13.67 mg/l) and the organism alone (14.33 mg/l) (Table 1). By encouraging microbial growth in soils, FYM enables organic matter to be mineralized by microorganisms, which releases nutrients into the crop gradually [44]. Primary P minerals, such as apatite, and secondary clay minerals, such as calcium, iron, and aluminum phosphates, are sources of P in soil and play a crucial role in preserving P availability and building via desorption and dissolution processes [70]. Applying FYM and biofertilizer causes the manure to break down and release macro and micronutrients, including P. It also dissolves the type of P that is not available by releasing organic acids throughout the breakdown process [70]. Because of the mineralization, solubilization, and translocation action of P-solubilizing bacteria through the generation of organic acid and proton extrusion, the use of biofertilizers has also increased the availability of P to plants [71,72]. Nahas [73] attributed the increase in solubilization of insoluble phosphate due to organic acids produced by microbes to the drop in soil pH, soil cations chelating, and competition with phosphate for adsorption sites in the soil solution. Kaur and Reddy [74] and Shahzad et al. [75] also observed improved soil P status with inoculation with phosphate-solubilizing bacteria resulting from higher alkaline phosphatase activity. Thus, the addition of FYM not only adds up to nutrient pools but also creates a favorable root zone environment for better root activity and nutrient uptake.

### 3.2 Effect of different treatments on the total carbohydrate and soluble protein contents of the leaves and seeds of groundnut plants

Mineral-solubilizing bacteria positively influence the biochemical content of plants by enhancing Zn and P uptake, which in turn improves chlorophyll synthesis, protein content, carbohydrate metabolism, antioxidant activity, phytohormone levels, nutrient use efficiency, and secondary metabolite production. This results in healthier plants with better growth, higher stress resistance, and improved overall productivity [76]. Therefore, the ability of the mineral-solubilizing bacterium *P. aeruginosa* to raise the carbohydrate and soluble protein content of the leaves of groundnut plants grown under diverse circumstances was evaluated at regular intervals of 30 days. The findings showed that the soluble protein and carbohydrate content of the leaves of T2, T3, and T4 plants recorded maximum values in comparison to the control (Table 2). On the 120th day of harvest, estimates of the groundnut plants’ seeds’ carbohydrate and soluble protein contents were also made. Additionally, the results revealed that T2, T3, and T4 plants’ groundnut seeds had the highest value when compared to the control (Table 3). This could be because of the common factor of all the three treatments “*P. aeruginosa* and FYM” provided vital nutrients for controlling biochemical activity in plants along with as nearly all of the enzymes and coenzymes involved in biochemical activity are activated by Zn [23] and P [77,78], respectively, through its mineral solubilizing ability. Studies have shown that MSB can increase the carbohydrate content in plants. For instance, a study on wheat, rice, and maize found that MSB increased the carbohydrate content in these crops [79,80]. This is likely since Zn and P play a crucial role in carbohydrate metabolism, and increased Zn and P availability can enhance photosynthesis and carbohydrate production. MSB has also been found to increase the protein content in plants. A study on groundnut plants found that zinc solubilizing bacteria (ZSB) increased the protein content in the plants, likely due to the increased availability of Zn, which is essential for protein synthesis [43,81]. Additionally, Zn-mobilizing plant growth-promoting rhizobacteria (PGPR) have been found to increase protein content in rice [82]. Additionally, threonine, an amino acid, has been shown to promote bacterial solubilization and plant uptake of nutrients, including P, which can lead to increased protein production [83]. According to Yousefi and Sadeghi [84], the application of FYM and vermicompost significantly increased the carbohydrate and protein content of wheat. This is likely due to the improved soil fertility and nutrient availability provided by FYM, which promotes healthy plant growth and development [85]. A similar finding was made by Prathiba and Siddalingeshwara [86], who investigated the impact of plant growth-promoting activity of *Bacillus subtilis* and *Pseudomonas fluorescence* as *Rhizobacteria* on seed quality of sorghum and observed that both strains were effective in enhancing seed quality, including seed germination, vigor index, and nutritional quality, including protein and carbohydrate contents. The favorable impact of *P. fluorescens* strain Psd on wheat production and nutritional quality was also evaluated by Sirohi et al. [87], who reported that seeds from plants collected from the strain Psd-infected soil had higher carbohydrate and protein contents than seeds from controls. Furthermore, Mathivanan et al. [88] evaluated the impact of PGPR (*Rhizobium + Pseudomonas + Bacillus*) on the nutritional value of groundnut (*A. hypogaea* L.) seedlings. Their findings showed that high biochemical content was observed in the groundnut seedlings grown using PGPR compared to uninoculated control. The current study thus shows the ability of *P. aeruginosa* to promote plant growth with effected the biochemical composition of the plants in conjunction with organic manure, which acted as a nutrient reservoir for its microbial activity.

**Table 2:** Effect of different treatments on the total carbohydrate and protein contents of the leaves of groundnut plants

Treatments	Total carbohydrate content of the plants (mg/g)				Protein Content of the Plants (mg/g)			
	30th	60th	90th	120th	30th	60th	90th	120th
T1	0.20 ± 0.03^c^	0.21 ± 0.02^c^	1.64 ± 0.11^c^	2.52 ± 0.19^b^	2.17 ± 0.11^b^	3.44 ± 0.22^a^	3.68 ± 0.17^c^	3.93 ± 0.28^b^
T2	0.32 ± 0.02^ab^	0.42 ± 0.04^a^	2.24 ± 0.16^b^	2.96 ± 0.21^ab^	2.25 ± 0.16^b^	3.85 ± 0.19^a^	3.94 ± 0.60^c^	4.09 ± 0.21^b^
T3	0.41 ± 0.09^a^	0.49 ± 0.05^a^	2.68 ± 0.24^a^	3.53 ± 0.31^a^	3.25 ± 0.34^a^	4.05 ± 0.71^a^	4.86 ± 0.21^a^	5.06 ± 0.30^a^
T4	0.32 ± 0.04^ab^	0.46 ± 0.07^a^	2.34 ± 0.17^ab^	3.08 ± 0.74^ab^	2.5 ± 0.38^b^	3.93 ± 0.64^a^	4.05 ± 0.77^ab^	4.88 ± 0.29^a^
T5	0.3 ± 0.06^b^	0.31 ± 0.08^b^	1.73 ± 0.36^c^	2.56 ± 0.39^b^	2.16 ± 0.18^b^	3.68 ± 0.37^a^	3.81 ± 0.74^c^	4.15 ± 0.44^b^
Treatments: 17.393***Days: 568.473***					Treatments: 14.139***Days: 81.132***			
Treatments × days: 2.652***					Treatments × days: 0.829***			

Values are mean ± SD of four replication samples in each group; *** indicates *p* < 0.001; ** indicates *p* < 0.01; and * indicates *p* < 0.05 versus control.

T1: control; T2: seeds treated with liquid inoculant; T3: seeds treated with liquid inoculant and the soil amended with FYM; T4: soil amended with FYM alone; T5: seeds treated with lignite (solid) based bioinoculant.

**Table 3:** Effect of different treatments on the carbohydrate and protein contents of the seeds

Treatments	Total carbohydrate (mg/g)	Soluble protein (mg/g)
T1	1.68 ± 0.27^c^	7.13 ± 0.77^c^
T2	2.22 ± 0.30^ab^	8.82 ± 0.16^b^
T3	2.56 ± 0.45^a^	10.1 ± 0.68^a^
T4	2.41 ± 0.19^ab^	8.88 ± 0.13^b^
T5	1.96 ± 0.44^bc^	8.08 ± 0.53^b^

Values are mean ± SD of four replication samples.

T1: control; T2: seeds treated with liquid inoculant; T3: seeds treated with liquid inoculant and the soil amended with FYM; T4: soil amended with FYM alone; and T5: seeds treated with lignite (solid)-based bioinoculant.

### 3.3 Effect of different treatments on the total chlorophyll content of the leaves of groundnut plants

**Figure 1:**
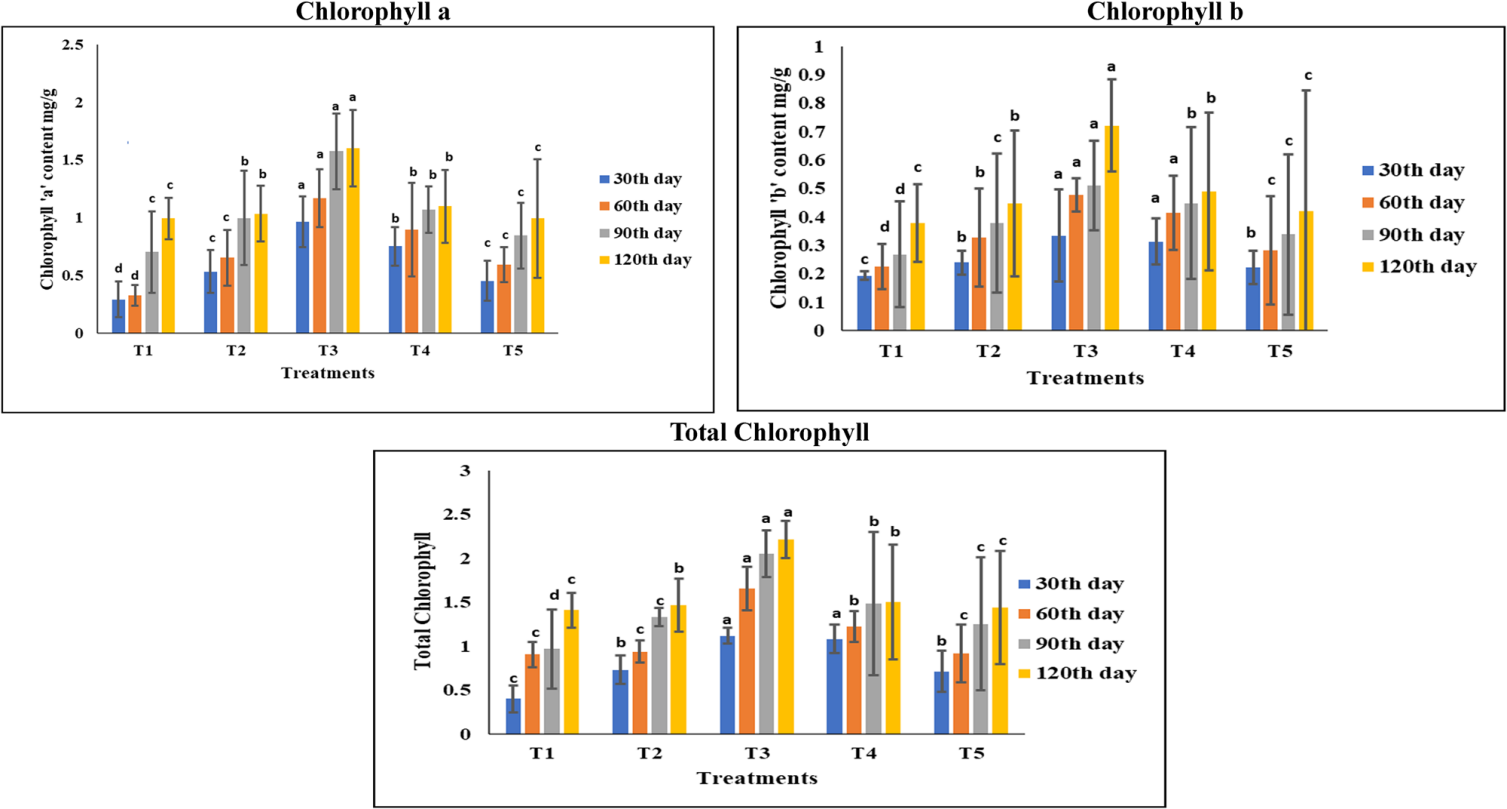
Effect of different treatments on the chlorophyll content of the leaves of groundnut plant. Values are mean ± SD of four replication samples. T1: control; T2: seeds treated with liquid inoculant; T3: seeds treated with liquid inoculant and the soil amended with FYM; T4: soil amended with FYM alone; and T5: seeds treated with lignite (solid)-based bioinoculant.

The ability of *P. aeruginosa* to promote plant growth by increasing the chlorophyll content (chlorophyll “a,” chlorophyll “b,” and total chlorophyll) of the leaves of groundnut plants raised under different treatments was assessed in the current experimental study at regular intervals of 30 days. The results demonstrated that, in comparison to the control, the groundnut leaves of T3 plants showed a significant (*p* < 0.05) increase in the levels of chlorophyll (chlorophyll “a,” chlorophyll “b,” and total chlorophyll) (Figure 1). Plants with low levels of Zn have less efficient photosynthesis [89]. It causes a 50–70% reduction in net photosynthesis in many plant species [90,91]. This decline in net photosynthesis in plants is ascribed to a reduction in Hill reaction activity, which may be brought on by a drop in chlorophyll concentration [92] and a disruption in the structure of the chloroplasts [25] in Zn-deficient plants. Under Zn-deficient conditions, Sharma et al. [92] reported that cauliflower leaves had significantly lower stomatal conductance, which resulted in a drop in CO_2_ supply, which in turn caused a considerable decrease in photosynthesis. In addition, Zn controls the synthesis of carotenoids and chlorophyll in plants, which is crucial for the efficient operation of the photosynthetic apparatus [93]. The application of MSB along with FYM can have a positive impact on the chlorophyll contents of plants. The chlorophyll content of plants has also been found to be influenced by the application of FYM and bioinoculants. Mean chlorophyll content values were higher with the application of 9.6 ton/ha of FYM [94]. Additionally, the use of bioinoculant has been found to promote the growth of beneficial microorganisms in the soil, which can help to increase the availability of nutrients, including sulfur, which is essential for chlorophyll formation [95]. Zn plays a crucial role in the synthesis of chlorophyll, and its deficiency can lead to reduced chlorophyll content and impaired photosynthesis [43]. Studies have demonstrated that the application of ZSB along with FYM can result in increased chlorophyll content, leading to improved photosynthesis and plant productivity [96,97]. The exact mechanisms behind this interaction are not fully understood, but it is believed that the ZSB’s ability to solubilize Zn and make it available to plants, combined with the nutrient-rich properties of FYM, contributes to the observed effects [79]. Research has shown that ZSB can increase the availability of Zn in the soil, which is an essential micronutrient for plant growth and development [98]. Studies have demonstrated that the application of phosphorus solubilizing bacteria (PSB) along with FYM can lead to a significant increase in chlorophyll content in plants. For instance, a study on okra plants found that the integrated use of PSB and FYM resulted in a 62% increase in chlorophyll content [99]. Similarly, another study on tomato plants showed that the inoculation of PSB strains, either singly or in combination, significantly increased the chlorophyll content of leaves [100]. The increased chlorophyll content in plants can be attributed to the ability of PSB to solubilize P in the soil, making it available to plants. The P is an essential nutrient for plant growth and development, and its availability can limit plant productivity. By solubilizing P, PSB can enhance plant growth and development, leading to increased chlorophyll content [101].

### 3.4 Analysis of percentage of oil content and the chemical characteristics of the oil extracted from the groundnut seeds harvested on the 120th day

**Figure 2:**
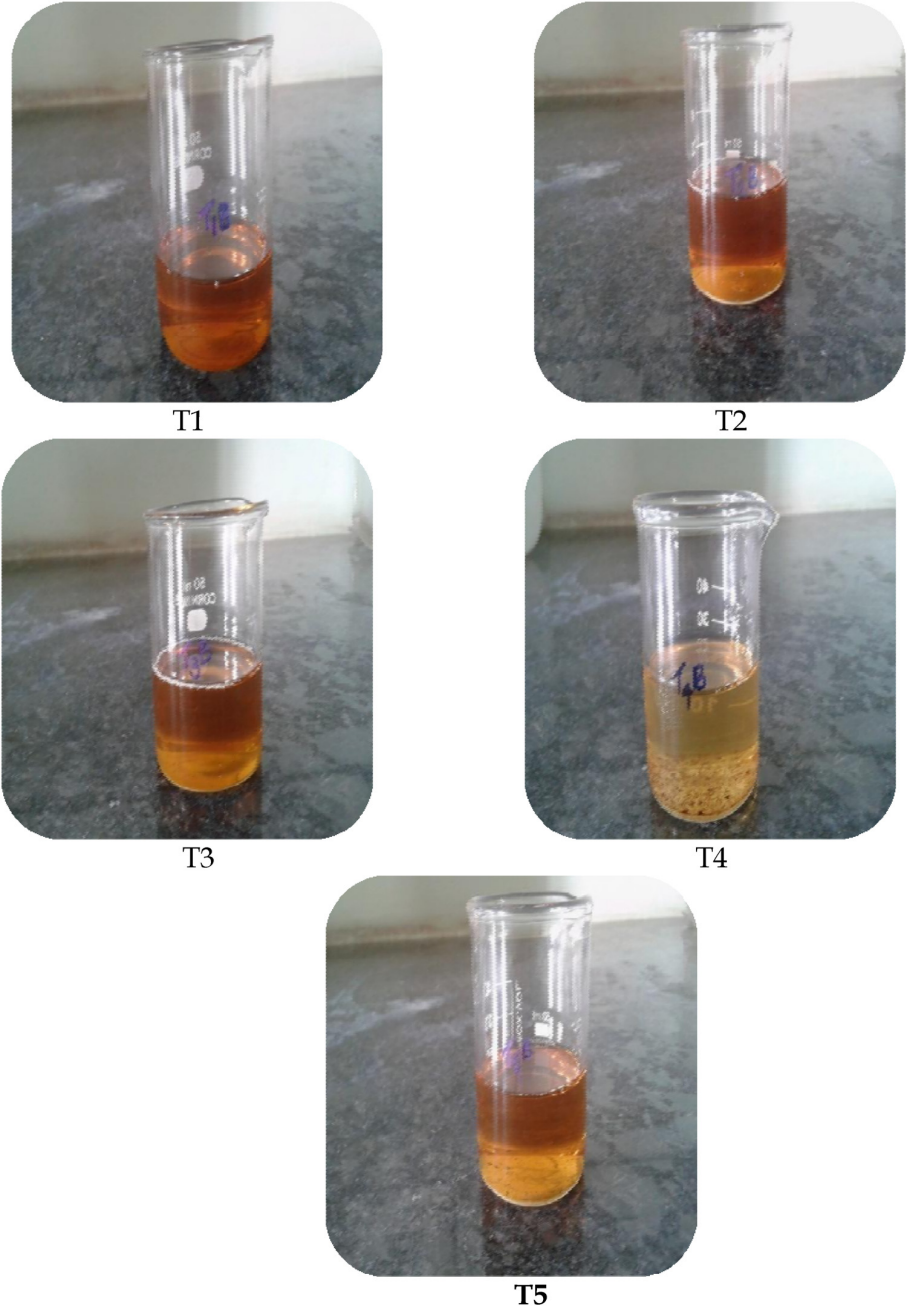
Oil extracted from the seeds of groundnut plants harvested on the 120th day. Oil from the seeds of the following treatments: T1: control; T2: seeds treated with liquid inoculant; T3: seeds treated with liquid inoculant and the soil amended with FYM; T4: soil amended with FYM alone; and T5: seeds treated with lignite (solid)-based bioinoculant.

Groundnut oil is an organic substance made from groundnut seeds that is known to taste and smell like its parent legume. In Table 4, the oil content and chemical properties of the oil extracted from the peanut seeds of the plants through various treatments are shown. It was a light-yellow tint and liquid at ambient temperature (Figure 2). The amount of crude oil in the peanut seeds of five different treatments ranged from 54.13 to 44.03% of the dry weight of the seeds (Table 4). The crude oil content varied significantly (*p* ≤ 0.05) among the seeds of the five different treatments. This may be attributable to the additive effects of liquid inoculant (*P. aeruginosa*) and organic manure, which would have enhanced seed oil content.

**Table 4:** Effect of different treatments on the oil percentage and the chemical characteristics of the oil extracted from the groundnut seeds

Treatments	Oil (%)	Iodine (mg KOH/g oil)	Saponification (mg KOH/g oil)	Free fatty acid (mg KOH/g oil)
T1	44.03 ± 1.34^c^	84.88 ± 0.77^c^	191.01 ± 0.91^d^	3.62 ± 0.51^a^
T2	46.05 ± 1.14^c^	86.92 ± 0.09^b^	194.36 ± 0.99^ab^	2.73 ± 0.38^bc^
T3	54.13 ± 1.54^a^	88.47 ± 0.44^a^	195.56 ± 1.05^a^	2.23 ± 0.53^c^
T4	50.08 ± 1.39^b^	87.17 ± 0.51^b^	192.85 ± 0.75^c^	3.12 ± 0.48^ab^
T5	45.1 ± 1.29^c^	86.24 ± 0.85^b^	193.06 ± 0.77^bc^	2.97 ± 0.30^ab^

Values are mean ± SD of four replication samples in each group.

Oil from the seeds of the following treatments: T1: control; T2: seeds treated with liquid formulation; T3: seeds treated with liquid formulation and the soil amended with FYM; T4: soil amended with FYM alone; and T5: seeds treated with lignite (solid)-based bioinoculant.

The degree of oil unsaturation can be gauged by the iodine value. For certain kinds of oil or fat, it remains constant. The iodine value is a valuable metric for analyzing the oxidative rancidity of oils since the higher the unsaturation, the higher the probability of the oils becoming rancid [102]. The findings of the iodine value of oil that was extracted from groundnut seeds under various treatments are shown in Table 4. The control had the least iodine content, at 84.88 mg KOH/g oil. A decrease in iodine value is a sign of lipid oxidation, and this could be because metallic ions were present and accelerated or allowed oxidation after hydroperoxide formed [103–107]. Oil from plant seeds grown using a combination of organic manure and liquid inoculant (T3) had the highest iodine content (88.47 mg KOH/g oil) when compared to organic manure alone (87.17 mg KOH/g oil). The iodine content of the oil extracted from the seeds of T2 and T5 plants yielded identical results.

The total quantity of milligrams of potassium hydroxide that reacts with one gram of sample is the saponification value, which is used to assess the amount of alkali reactive groups in fats and oils [101]. In comparison to the control (191.01 mg KOH/g), the oil from the seeds of T3 plants had a greater saponification value (195.56 mg KOH/g). Similar outcomes were seen in the seeds of the T2 and T5 plants. Higher saponification values indicate a higher percentage of lower fatty acids since they are inversely related to the average molecular weight or chain length of the fatty acids [108]. Due to the oil’s high saponification value, its potential use in the saponification sector may be suggested.

The acid number or acid value refers to the quantity of free fatty acids. It specifies the oil’s functional characteristics, shelf life, nutritional value, and flavors [109]. So, the amount of free fatty acids determines the oil’s quality. The results for the oil’s free fatty acid content are displayed in Table 4. Compared to control plants (3.62 g oleic acid), the oil recovered from the seeds of T3 plants contained the least quantity of free fatty acid (2.23 g). In comparison to other treatments, the oil extracted from the seeds of the T2 and T5 plants produced comparable results. The stability of the products is shown by the low free fatty acid content of the oil [110]. If oil is stored for a long time, its unpleasant flavor and odor are caused by the existence of free fatty acids and other fatty components [111]. Because the sample of oil obtained from various treatments had a pleasant smell. This can be caused by the low levels of free fatty acids present. The oil’s suitability for human ingestion increases with a decrease in free fatty acid content [112].

As shown by the results, the application of liquid inoculant along with organic manure (T3) had a significant impact on the oil yield and its chemical characteristics, which may be attributable to the availability of vital nutrients like Zn and P due to *P. aeruginosa*’s activity in mineral solubilizing. Similar results were observed by Radwan and Awad [113] who discovered that applying biofertilizer (*Azospirillum* sp. and *Pseudomonas* sp.) together with organic amendments significantly increased the oil yield and quality of peanut seeds in comparison to chemical fertilizer treatment. Safwat and Badran [114] additionally observed that the cumin plants’ essential oil production was increased when organic amendments mixed with *Azotobacter* and *Bacillus megaterium* were applied. Mekki et al. [115] also stated that soybean plants fertilized with biofertilizers in addition to organic manure had higher soybean seed oil levels. The largest amount of essential oil was produced when fennel plants were fertilized with organic manure and biofertilizers (*Azotobacter* and *Azospirillum*), according to Azzaz et al. [116]. As a result, in the current study, liquid inoculants and organic manure improved the oil content of the seeds of groundnut plants by making vital nutrients available to the plants through their action to promote plant growth.

### 3.5 Analysis of characteristics of soil before and after treatments

The addition of mineral-solubilizing bacteria to field soil in the form of a liquid bioinoculant would alter the soil’s characteristics because of the microorganisms’ ability to promote plant growth. Therefore, both before and after treatments, the parameters of the soil, such as pH, EC, CaCO_3_, organic carbon, and mineral content, including NPK and Zn, were investigated in this study. The results showed that all treatments significantly improved the soil’s properties in comparison to the control (Table 5).

**Table 5:** Characteristics of the soil before and after treatments

Treatments	pH	EC (dS/m)	N (kg/ha)	P (kg/ha)	K (kg/ha)	Zn (mg/kg)	CaCO_3_ (%)	SOC (%)
Before treatment	8.3 ± 0.17^a^	2.88 ± 0.32^a^	273.75 ± 2.06^f^	20.1 ± 2.12^e^	182.25 ± 4.5^e^	1.53 ± 0.10^d^	2.53 ± 0.20^a^	0.39 ± 0.03^d^
T1	8.08 ± 0.15^ab^	1.35 ± 0.15^b^	417.00 ± 2.94^e^	29.6 ± 0.81^d^	204.75 ± 4.27^d^	2.09 ± 0.78^cd^	1.13 ± 0.28^c^	0.75 ± 0.02^c^
T2	7.8 ± 0.25^bc^	0.37 ± 0.09^d^	478.75 ± 1.70^c^	37.33 ± 1.58^c^	218.00 ± 4.89^c^	5.38 ± 0.97^b^	1.3 ± 0.24^c^	0.84 ± 0.04^ab^
T3	7.5 ± 0.31^c^	0.30 ± 0.05^d^	788.25 ± 1.25^a^	59.65 ± 1.71^a^	346.5 ± 4.72^a^	7.24 ± 0.83^a^	1.7 ± 0.14^b^	1.91 ± 0.05^a^
T4	7.9 ± 0.22^b^	0.35 ± 0.10^d^	483.75 ± 2.62^b^	47.83 ± 1.22^b^	231.5 ± 2.51^b^	6.79 ± 1.26^a^	1.3 ± 0.40^c^	0.89 ± 0.06^a^
T5	8 ± 0.18^ab^	0.93 ± 0.14^c^	432.00 ± 2.82^d^	33.15 ± 1.49^d^	207.25 ± 4.11^d^	3.22 ± 0.99^c^	1.23 ± 0.09^c^	0.81 ± 0.08^bc^

Values are mean ± SD of four replication samples.

T1: control; T2: seeds treated with liquid formulation; T3: seeds treated with liquid formulation and the soil amended with FYM; T4: soil amended with FYM alone; and T5: seeds treated with lignite (solid)-based bioinoculant.

The pH of the soil, which represents the degree of acidity in the soil, has a big impact on microbial activity, plant nutrition availability, and even soil aggregate durability. The pH of the soil in the T3 plot was 7.5, which is lower than in the other treatments. This suggests that the addition of FYM and liquid bioinoculant affected the physical, chemical, and biological processes as well as the characteristics of the soil [117,118]. The pH of all the treated soils slightly decreased after the last groundnut plant was harvested. This could be because the bioinoculant *P. aeruginosa* produced organic acids, which led to a drop in pH, which is thought to be the main mechanism of metal solubilization. Because of the soil’s natural ability to act as a buffer in this experiment, it was discovered that soil pH decline was far lower than in the culture medium. Similar to this, Son et al. [119] showed that the main source of mineral (such as Zn and P) solubilization was the acidification of the culture by bacteria; however, a high level of metal solubilization compared to *in vitro* may not be possible in soil because most soils have a great pH buffering capacity. Lakshmi et al. [120] also observed similar results, i.e., the highest reduction in soil pH with the combined application of biofertilizers.

Soil EC has a direct impact on plants growing in the soil, it is a valuable indication for controlling agricultural systems [121] as the quantity of moisture that soil particles can hold varies with soil EC. Good soil health is often indicated by an EC range of 0–1 dS/m [122]. According to the current study, all of the inoculant-treated soil had an EC range of 0.3–1.3, which is acceptable and demonstrates the soil’s fertility [123].

The T3 plot showed a drop in EC contents, which is consistent with findings from Sushila et al. [124], who proposed that applying biofertilizers to saline soil would reduce soil salinity because the biofertilizers activate microorganisms in soil and increase the production of the enzyme dehydrogenase led to decrease the soil salinity compared with control. Additionally, Shaban and Attia [125] demonstrated that the values of EC dropped with an increase in mineral fertilizer in combination with biofertilizer, as compared to mineral fertilizers alone.

SOC plays a significant role in improving soil quality by improving ion exchange capacity, supporting biological component function, reducing bulk density, increasing water holding capacity, and increasing macro- and micronutrient availability [126,127]. SOC concentration greater than 1.7% indicates fertile soil [128]. In the current study, a T3 plot with a SOC concentration of 1.9% demonstrated that biofertilizers both directly and indirectly promote SOC accrual through microbial biomass turnover, the secretion of organic extracellular polymeric substances, or promoting plant growth through nutrient solubilization, associations, hormonal changes, etc. [129].

The physical and chemical properties of soil are impacted by the presence of CaCO_3_. Excessive lime concentration might hinder root penetration and water circulation. Within the soil’s fertile range, all the plots had CaCO_3_ concentrations ranging between 1 and 2.5% [123]. As a result, all of the inoculant-treated soil has optimal pH, EC, SOC, and CaCO_3_ content. Additionally, plant nutrients including N, P, K, and Zn were found to be within the fertile range of >480, >56, >280, and >0.6 kg/ha [123].

In terms of soil characteristics, the incorporation of mineral solubilizing bacteria with organic manure (T3) performed better than the other treatments, although there is no significant difference in the properties of the soil treated with different treatments when compared to liquid inoculant (T2), organic manure (T4), and control (T1) (Table 5). This could be a result of the advantageous effects of organic manures on nutrient availability. They accomplish this by directly or indirectly influencing the chemical processes that alter soil microbial activity and nutrients. These findings also correspond with the study by Mengel et al. [130], who stated that adding organic manure and biofertilizer promoted the synthesis of chemicals that control growth, enhancing the physico-chemical characteristics, microbial activity, and soil’s ability to retain water. Mahapatra et al. [131] also observed that bacterial biofertilizers are crucial for boosting root accessibility to soil components including N, P, K, and Zn, which promotes rhizosphere biocontrol. Thus, the current study observed that the liquid inoculant (*P. aeruginosa*) and organic manure had a positive impact on the availability of N, P, K, and Zn as well as other soil parameters, demonstrating the essential role of this organism in the transformation reaction of vital nutrients in the soil through its numerous plant growth-promoting activities, such as mineral solubilization and phytohormone synthesis. Regenerative agriculture practices involving the incorporation of organic matter (FYM application and residue retention) among others [132] can strongly influence the soil physicochemical parameters and biological properties and enhance soil fertility by stimulating microbial abundance and nutrient cycling and regulating SOC availability [99]. The application of organic matter (i.e., FYM) has been shown to improve soil physiochemical conditions [60] alongside stimulating a positive microbial link between nutrient availability [133], the regulation of SOC, and moisture.

## 4 Conclusion

The current study reveals that *P. aeruginosa* can effectively improve *A. hypogaea* L.’s biochemical profile by providing essential minerals (Zn and P) through the solubilization process. The incorporation of PVP proved to be a potential ingredient in *P. aeruginosa* liquid formulations as it protects bacteria by reducing their mortality and prolonging their shelf life. Liquid formulant supplementation in conjunction with FYM is also very beneficial because it provides the bioinoculant with a carbon and energy source, which improves nutrient availability, prolongs nutrient retention in the soil, and increases soil carbon sequestration, which reduces climate change by absorbing and storing atmospheric carbon dioxide. Thus, the study implies that crops cultivated using liquid bioinoculants will be more nutritious, leading to healthier products for consumers, as they boost nutrient availability and uptake. This kind of bioinoculant has the potential to replace chemical fertilizers, thereby reducing their adverse ecological impacts and leading to more sustainable agricultural practices. This is consistent with customers’ growing demands for environmentally friendly and sustainable food production methods. Overall, bioinoculants generally aid in the advancement of a more sustainable agricultural system that is advantageous to both food producers and consumers.

## References

[1] Mengel K, Kirkby EA. Principles of plant nutrition. Ann Bot. 2004;93(4):479–80.

[2] Monib AW, Alimyar O, Mohammad MU, Akhundzada MS, Niazi P. Macronutrients for plants growth and human health. J Res Appl Sci Biotech. 2023;2:268–9.

[3] Jan AU, Hadi F, Ditta A, Suleman M, Ullah M. Zn-induced anti-oxidative defense and osmotic adjustments to enhance drought stress tolerance in sunflower (Helianthus annuus L.). Env Exp Bot. 2022;193:104682.

[4] Johnson VJ, Mirza A. Role of macro and micronutrients in the growth and development of plants. Int J Curr Microbiol Appl Sci. 2020;9:576–87.

[5] Barker AV, Pilbeam DJ, (Eds.). Handbook of plant nutrition. Boca Raton: CRC Press; 2015.

[6] Voortman RL, Bindraban PS. Beyond N and P: Toward a land resource ecology perspective and impactful fertilizer interventions in sub-Saharan Africa. VFRC Report 2015/1. Washington, DC: Virtual Fertilizer Research Center; 2015.

[7] Monreal CM, DeRosa M, Mallubhotla SC, Bindraban PS, Dimkpa CO. Nanotechnologies for increasing the crop use efficiency of fertilizer-micronutrients. Biol Fertil Soils. 2016;52(3):423–37.

[8] Fageria NK, Baligar VC, Li YC. The role of nutrient efficient plants in improving crop yields in the twenty first Century. J Plant Nutr. 2008;31:1121–57.

[9] Baligar VC, Fageria NK, He ZL. Nutrient use efficiency in plants. Commun Soil Sci Plant Anal. 2001;32:921–50.

[10] Alloway BJ. Zn in soils and crop nutrition. International Zn Association, Brussels, Belgium; 2008.

[11] Otieno EO, Mucheru-Muna MW, Kifuko-Koech MN, Kamau CN, Ndung’u-Magiroi KW H, Mogaka H, et al. Strategic research in the domain of secondary nutrients, micronutrients, liming and 4R stewardship in sub-Saharan Africa: Review. Environ Chall. 2024;16:100960.

[12] Graham RD. Micronutrient deficiencies in crops and their global significance. In: Alloway BJ, editors. Micronutrient deficiencies in global crop production. Dordrecht: Springer; 2008.

[13] Zaheer IE, Ali S, Saleem MH, Ali M, Riaz M, Javed S, et al. Interactive role of Zn and iron lysine on Spinacia oleracea L. growth, photosynthesis and antioxidant capacity irrigated with tannery wastewater. Physiol Mol Biol. 2020a;26(12):2435–52. 10.1007/s12298-020-00912-0 PMC7772129 33424157

[14] Yang M, Li Y, Liu Z, Tian J, Liang L, Qiu Y, et al. A high activity Zn transporter OsZIP9 mediates Zn uptake in rice. Plant J. 2020;103:1695–709. 10.1111/tpj.14855 32449251

[15] Alsafran M, Usman K, Ahmed B, Rizwan M, Saleem MH, Al Jabri H. Understanding the phytoremediation mechanisms of potentially toxic elements: A proteomic overview of recent advances. Front Plant Sci. 2022;13:881242. 10.3389/fpls.2022.881242 PMC9134791 35646026

[16] Zlobin I. Current understanding of plant Zn homeostasis regulation mechanisms. Plant Physiol Biochem. 2021;162:327–35. 10.1016/j.plaphy.2021.03.003 33714765

[17] Hamzah Saleem M, Usman K, Rizwan M, Al Jabri H, Alsafran M. Functions and strategies for enhancing Zn availability in plants for sustainable agriculture. Front Plant Sci. 2022;13:1033092. 10.3389/fpls.2022.1033092 PMC9586378 36275511

[18] Nandal V, Solanki M. Zn as a vital micronutrient in plants. J Microbiol Biotechnol Food Sci. 2021;11:e4026.

[19] Souvik N, Manoranjan O, Tapu Raihan G, Partha G, Melepurath D. Dendrite growth inhibition in a V6O13 nanorods based non-aqueous Zn-ion battery by a scalable polycarbazole at Carbon nanotubes overlayer. Compos B Eng. 2023;252:110516.

[20] Sharma SB, Sayyed RZ, Trivedi MH, Gobi TA. Phosphate solubilizing microbes: sustainable approach for managing P deficiency in agricultural soils. SpringerPlus. 2013;2:587. 10.1186/2193-1801-2-587 PMC4320215 25674415

[21] Marschner H, Cakmak J. High light intensity enhances chlorosis and necrosis inleaves of Zn, potassium, and magnesium deficient bean (Phaseolus vulgaris L.) plants. J Plant Physiol. 1989;134:924–34.

[22] Faisal N, Sundas A, Faiza W, Najeeb A, Rashid M, Sadia B, et al. P (P) and Zn (Zn) nutrition constraints: A perspective of linking soil application with plant regulations. Env Exp Bot. 2024;226:105875.

[23] Mousavi SR. Zn in crop production and interaction with P. Aust J Basic Appl Sci. 2011;5(9):1503–9.

[24] Taheri N, Heidari H, Yousefi K, Mousavi SR. Effect of organic manure with P and Zn on yield of seed potato. Aust J Basic Appl Sci. 2011;5(8):775–80.

[25] Brown PH, Cakmak IQ, Zhang Q. Form and function of Zn in plants. In: Robson AD, editors. Zn in soils and plants. Dordrecht, Boston, London: Kluwer Academic Publishers; 1993. p. 90–106.

[26] Taiz L, Zeiger E. Plant physiology: mineral nutrition. Redwood City: The Benjamin Cummings Publishing Co.; 1991. pp. 100–19.

[27] Marschner M. Mineral nutrition of higher plants. 2nd edn. London: Academic Press; 1995. p. 200–55.

[28] Outten CE, O’Halloran TV. Femtomolar sensitivity of metallo regulatory protein controlling Zn homeostasis. Science. 2001;292:2488–92. 10.1126/science.1060331 11397910

[29] Pandey N, Pathak GC, Sharma CP. Zn is critically required for pollen function and fertilization in lentils. J Trace Elem Med Biol. 2006;20:89–96. 10.1016/j.jtemb.2005.09.006 16785048

[30] Bojovic B, Stojanovic J. Chlorophyll and carotenoid content in wheat cultivars as a function of mineral nutrition. Arch Biol Sci. 2005;57(4):283–90.

[31] Odoemelam SA. Proximate composition and selected physicochemical properties of the seeds of African Oil Bean (Pentaclethra marcrophylla). Pak J Nutr. 2005;4:382–3.

[32] Morrison WH, Hamilton RJ, Kalu C. Sunflower seed oil. In: Hamilton RJ, editor. Developments in oils and fats. Glasgow: Blackie Academic and Professional; 1995. p. 132–52.

[33] Omidian A, Seyadat SA, Naseri R, Moradi M. Effects of Zn-sulfate foliar onyield, oil content and seed protein of four cultivars of canola. Iranian. J Agric Sci. 2012;14:26–8.

[34] Singh AL. Mineral nutrition of groundnut. In: Helantaranjan A, editor. Advances in plant physiology. Jodhpur, India: Scientific Publishers; 1999. p. 161–200.

[35] Singh AL, Basu MS, Singh NB. Mineral disorders of groundnut, National Research centre for groundnut. Junagadh, India: National Research centre for groundnut (ICAR); 2004. p. 85.

[36] Alloway BJ. Zn in soils and plant nutrition. 2nd edn. Brussels, Belgium and Paris, France: International Zn Association and International Fertilizer Industry Association; 2008. p. 139.

[37] ILZRO. Zn in crop nutrition. International Lead Zn Research Organisation Inc, Research Triangle Park, Durham, North Carolina; 1975. p. 54.

[38] Klug A, Rhodes D. “Zn fingers”: A novel protein motif for nucleic acid recognition. Trends Biochem Sci. 1987;12:464–9.

[39] Romheld V, Marschner H. Micronutrients in agriculture. Soil Science Society of America Book Series, No 4, Published by: Soil Science Society of America, Inc., Madison, Wisconsin, USA. 2nd edn. 1991. p. 297–28.

[40] Kumar P, Kaushal N, Dubey RC. Isolation and identification of plant growth promoting rhizobacteria (Pseudomonas sp.) and their effect on growth promotion of Lycopersicon esculentum L. Academ. Arena. 2015;7(5):44–51.

[41] Sunithakumari K, PadmaDevi S, Vasandha S. Zn solubilizing bacterial isolates from the agricultural fields of Coimbatore, Tamil Nadu, India. Curr Sci. 2016;110:196–205.

[42] Devi SNP, Kumari KS, Vasandha S. Assessment of competence of the Pseudomonas aeruginosa to solubilize insoluble form of Zn under various cultural parameters. Arab J Sci Eng. 2016;41:2117–21.

[43] Devi SP, Ranjithkumar R, Djearamane S, Tey LH, Wong LS, Kayarohanam S, et al. Organic Remobilization of Zn and P availability to plants by application of mineral solubilizing bacteria Pseudomonas aeruginosa. Heliyon. 2023;9(11):e22128. 10.1016/j.heliyon.2023.e22128 PMC10694168 38053868

[44] Nithya K. Studies on solubilization of minerals by soil microorganisms (B.Sc., Agri., thesis) Department of Agricultural Microbiology, Agricultural College and research institute, Tamil Nadu Agricultural University; 2008.

[45] Lindsay L, Norvell WA. Development of DTPA soil test for Zn, iron, manganese and copper. Soil Sci Am J. 1978;42:421–8.

[46] Olsen SR, Cole CV, Watanabe S, Dean LA. Estimation of available P in soils by extraction with sodium bicarbonate. US Dept Agr Circ. 1954;939:19.

[47] Hedge JE, Hofreiter BT. Methods in carbohydrate chemistry. In: Whistler RL, Be Miller JN, editor. Vol. 17, New York: Academic Press; 1962. p. 420.

[48] Bradford MM. A rapid and sensitive method for the quantitation of microgram quantities of protein utilizing the principle of protein-dye binding. Anal Biochem. 1976;72:248–54. 10.1016/0003-2697(76)90527-3 942051

[49] Arnon DI. Copper enzymes in isolated chloroplast. Plant Physiol. 1949;24:1. 10.1104/pp.24.1.1 PMC437905 16654194

[50] Anjani MK. Extraction and characterization of crude and refined groundnut (Arachis hypoagae, L.) oil. Int J Chem stud. 2017;5:2031–3.

[51] AOAC (Association of Official Analytical Chemist), Official method of Analysis. Washington D.C.; 1984.

[52] Sabir H, Rownok J, Ashraful A, Khodeza K, Sharif MA. Study on physic-chemical properties of edible oils available in Bangaladesh local market. Arch Curr Res Int. 2016;6:1–6.

[53] Birnin-Yauri UA, Garba S. Comparative studies of some physico-chemical properties of Baobab, Vegetables, Peanut and Palm oils. Niger J Basic Appl Sci. 2011;19:64–7.

[54] Jackson ML. Soil chemical analysis. Prentice Hall of India, New Delhi, India; 1973.

[55] Dreleimanis A. Quantities gasometric determination of calcite and dolomite by using Chittick apparatus. J Sediment Pet. 1962;32(3):20–9.

[56] Walkley A, Black IA. An examination of Degtjareff method for determining soil organic matter and a proposed modification of the chromic acid titration method. Soil Sci. 1934;37:29–37.

[57] Vogel AI. A textbook of quantitative inorganic analysis. Chapter X. New York: Longman Inc.; 1961. p. 312.

[58] Mahendra S, Ruhal DS, Narendra S. Zn availability in sodic soils reclaimed partially to different ESP levels. J Indian Soil Soc. 1993;41(2):256–60.

[59] Fasim F, Ahmed N, Parsons R, Gadd GM. Solubilization of Zn salts by a bacterium isolated from the air environment of a tannery. FEMS Microbiol Lett. 2002;213(1):1–6. 10.1111/j.1574-6968.2002.tb11277.x 12127480

[60] Mucheru-Muna M, Mugendi D, Pypers P, Mugwe J, James K, Vanlauwe B, et al. Enhancing maize productivity and profitability using organic inputs and mineral fertilizer in central Kenya small-hold farms. Exp Agric. 2014;50:250–69.

[61] Mugwe J, Mugendi D, Mucheru-Muna M, Odee D, Mairura F. Effect of selected organic materials and inorganic fertilizer on the soil fertility of a humic nitisol in the central highlands of Kenya. Soil Use Manag. 2009;25:434.

[62] Kihanda FM, Warren GP, Micheni AN. Effects of manure application on crop yield and soil chemical properties in a long-term field trial in semi-arid Kenya. In: Bationo A, Waswa B, Kihara J, Kimetu J, editors. Advances in integrated soil fertility management in Sub-Saharan Africa: Challenges and opportunities. Dordrecht, The Netherlands: Springer; 2007. p. 471–86.

[63] Gautam A, Sekaran U, Guzman J, Kovács P, Hernandez JLG, Kumar S. Responses of soil microbial community structure and enzymatic activities to long-term application of mineral fertilizer and beef manure. Env Sustain Indic. 2020;8:100073.

[64] Tang H, Li C, Xiao X, Shi L, Cheng K, Wen L, et al. Effects of short-term manure nitrogen input on soil microbial community structure and diversity in a double-cropping paddy field of Southern China. Sci Rep. 2020;10:13540. 10.1038/s41598-020-70612-y PMC7419555 32782287

[65] Babin D, Deubel A, Jacquiod S, Sørensen SJ, Geistlinger J, Grosch R, et al. Impact of long-term agricultural management practices on soil prokaryotic communities. Soil Biol Biochem. 2019;129:17–28.

[66] Lladó S, Baldrian P. Community-level physiological profiling analyses show potential to identify the copiotrophic bacteria present in soil environments. PLoS ONE. 2017;12:e0171638. 10.1371/journal.pone.0171638 PMC5295708 28170446

[67] Senthil Kumar PS, Aruna Geetha S, Savithri P, Jagadeeswaran R, Ragunath KP. Effect of Zn enriched organic manures and Zn solubilizer application on the yield, curcumin content and nutrient status of soil under turmeric cultivation. J Appl Hortic. 2004;6(2):82–6.

[68] Kushwaha P, Kashyap PL, Pandiyan K, Bhardwaj AK. Zn-solubilizing microbes for sustainable crop production: current understanding, opportunities, and challenges. In: Solanki MK, Kashyap PL, Kumari B, editors. Phytobiomes: Current insights and future vistas. Singapore: Springer; 2020. p. 281–98.

[69] Nitu R, Rajinder K, Sukhminderjit K. Zn solubilizing bacteria to augment soil fertility – A comprehensive review. Int J Agric Sci Vet Med. 2020;8:38–44.

[70] Mitran T, Mani PK. Effect of organic amendments on rice yield trend, P use efficiency, uptake, and apparent balance in soil under long-term rice-wheat rotation. J Plant Nutr. 2017;40(9):1312–22.

[71] Marra LM, de Oliveira SM, Soares CRFS, de Souza Moreira FM. Solubilisation of inorganic phosphates by inoculants strains from tropical legumes. Sci Agricola. 2011;68:603–9.

[72] Gupta G, Panwar J, Jha P. Natural occurrence of Pseudomonas aeruginosa, a dominant cultivable Diazotrophic endophytic bacterium colonizing Pennisetum glaucum (L) R. Br. Appl Soil Ecol. 2013;64:252–61.

[73] Nahas E. Factors determining rock phosphate solubilization by microorganism isolated from soil. World J Microbiol Biotechnol. 1996;12:18–23. 10.1007/BF00327716 24415416

[74] Kaur G, Reddy MS. Influence of P-solubilizing bacteria on crop yield and soil fertility at multi locational sites. Euro J Soil Biol. 2014;61:35–40.

[75] Shahzad SM, Khalid A, Arif MS, Riaz M, Ashraf M, Iqbal Z. Co-inoculation integrated with P-enriched compost improved nodulation and growth of chickpea (Cicer arietinum L.) under irrigated and rainfed farming systems. Biol Fertil Soils. 2014;50:1–12.

[76] Andrade DLA, Santos CHB, Frezarin ET, Sales LR, Rigobelo EC. Plant growth-promoting rhizobacteria for sustainable agricultural production. Microorganisms. 2023 Apr;11(4):1088. 10.3390/microorganisms11041088 PMC10146397 37110511

[77] Bisson P, Cretenet M, Jallas E. Nitrogen, P and potassium availability in the soil physiology of the assimilation and use of these nutrients by the plant. In: Constable GA, Forrester NW, editros. Challenging the Future: Proceedings of the World Cotton Research Conference-1, Brisbane Australia, February 14–17. Melbourne: CSIRO; 1994. p. 15–124.

[78] Rodriguez D, Zubillaga MM, Ploschuck E, Keltjens W, Goudriaan J, Lavado R. Leaf area expansion and assimilate prediction in sunflower growing under low P conditions. Plant Soil. 1998;202:133–47.

[79] Upadhayay VK, Singh AV, Khan A. Cross talk between Zn-solubilizing bacteria and plants: a short tale of bacterial-assisted Zn biofortification. Front Soil Sci. 2022;1:788170.

[80] Silva LI, Pereira MC, Carvalho AM, Buttrós VH, Pasqual M, Dória J. Phosphorus-solubilizing microorganisms: a key to sustainable agriculture. Agriculture. 2023;13(2):462.

[81] Timofeeva AM, Galyamova MR, Sedykh SE. Bacterial siderophores: classification, biosynthesis, perspectives of use in agriculture. Plants. 2022;11(22):3065. 10.3390/plants11223065 PMC9694258 36432794

[82] Singh S, Chhabra R, Sharma A, Bisht A. Harnessing the power of Zn-solubilizing bacteria: a catalyst for a sustainable agrosystem. Bacteria. 2024;3(1):15–29.

[83] Pantigoso HA, Manter DK, Fonte SJ, et al. Root exudate-derived compounds stimulate the P solubilizing ability of bacteria. Sci Rep. 2023;13:4050. 10.1038/s41598-023-30915-2 PMC10006420 36899103

[84] Yousefi AA, Sadeghi M. Effect of vermicompost and urea chemical fertilizers on yield and yield components of wheat (Triticum aestivum) in the field condition. Intl J Agri Crop Sci. 2014;7(12):1227–30.

[85] Agrawal SB, Anoop Singh AS, Gaurav Dwivedi GD. Effect of vermicompost, farm yard manure and chemical fertilizers on growth and yield of wheat (Triticum aestivum L. var. HD 2643). Plant Arch. 2003;3:9–14.

[86] Prathibha KS, Siddalingeshwara KG. Effect of plant growth promoting Bacillus subtilis and Pseudomonas fluorescence as rhizobacteria on seed quality of sorghum. Int J Curr Microbiol App Sci. 2013;2(3):11–8.

[87] Sirohi G, Upadhyay A, Srivastava PS, Srivastava S. PGPR mediated Zn biofertilization of soil and its impact on growth and productivity of wheat. J Soil Sci Plant Nutr. 2015;15:202–16.

[88] Mathivanan S, Chidambaram ALA, Sundaramoorthy P, Baskaran L, Kalaikandhan R. The effect of plant growth promoting rhizobacteria on groundnut (Arachis hypogaea L.) seed germination and biochemical constituents. Int J Curr Res Aca Rev. 2014;2(9):187–94.

[89] Shrotri CK, Rathore VS, Mohanty P. Studies on photosynthetic electron transport, photophosphorylation and CO2 fixation in Zn deficient leaf cells of Zea mays L. J Plant Nutr. 1981;3:945–54.

[90] Pearson JN Z, Rengel Z. Genotypic differences in the production and partitioning of carbohydrates between roots and shoots of wheat grown under Zn or manganese deficiency. Ann Bot. 1997;80:803–8.

[91] Alloway BJ. Zn in soils and crop nutrition. International Zn Association (IZA)Publications, Brussels: Belgium. 2004. p. 1–116.

[92] Sharma PN, Kumar N, SBisht SS. Effect of Zn deficiency on chlorophyll content, photosynthesis and water relations of cauliflower plants. Photosynthetica. 1994;30:353–9.

[93] Aravind P, Prasad MNV. Zn protects chloroplasts and associated photochemical functions in cadmium-exposed Ceratophyllum demersum L., a freshwater macrophyte. Plant Sci. 2004;166:1321–27.

[94] Farid H, Saied El S, Amany Abdel Mohsen R, Doaa MA. The combined effects of farm yard manure and boron application on growth, and oil quality of Canola grown under newly reclaimed soils. Oil Crop Sci. 2024;9(1):53–9.

[95] Chaudhary P, Singh S, Chaudhary A, Sharma A, Kumar G. Overview of biofertilizers in crop production and stress management for sustainable agriculture. Front Plant Sci. 2022;23(13):930340. 10.3389/fpls.2022.930340 PMC9445558 36082294

[96] Kamran S, Shahid I, Baig DN, Rizwan M, Malik KA, Mehnaz S. Contribution of Zn solubilizing bacteria in growth promotion and Zn content of wheat. Front Microbiol. 2017;21(8):2593. 10.3389/fmicb.2017.02593 PMC5743011 29312265

[97] Chanchal AK, Singh M, Pradhan AK, Kumar S, Beura K, Singh P. Changes in soil Zn fractions upon inoculation of Zn solubilizing bacteria (ZnSB) under rice rhizospheric soil. Commun Soil Sci Plant Anal. 2024;55(15):2322–38.

[98] Bolo P, Mucheru-Muna MW, Mwirichia RK, Kinyua M, Ayaga G, Kihara J. Influence of farmyard manure application on potential Zn solubilizing microbial species abundance in a ferralsol of Western Kenya. Agriculture. 2023;13(12):2217.

[99] Rahim N, Yasin Z, Tahir M, Majeed A, Yaqub A, Mahmood B. Rock phosphate and manures with P solubilizing bacteria increases the growth, yield and P uptake of okra (Abelmoschus esculentus). J Appl Res Plant Sci. 2023;5:111–8.

[100] Wu F, Li J, Chen Y, Zhang L, Zhang Y, Wang S, et al. Effects of phosphate solubilizing bacteria on the growth, photosynthesis, and nutrient uptake of Camellia oleifera Abel. Forests. 2019;10(4):348.

[101] Pyone A, Pechrada P, Saowanuch T. Effect of P solubilizing bacteria on soil available P and growth and yield of sugarcane. Walailak J Sci Tech (WJST). 2021;18(12):1–9.

[102] Sadasivam S, Manickam A. Biochemical methods. 3rd edn. New Delhi, India: New Age International Publishers; 2008.

[103] Joseph AF. Measuring flavour deterioration of fats, oils and foods. New York: General Food Cooperation Technical Center; 1977. p. 1–7.

[104] Tanlor SL, Beg CM, Shoptangh NH, Traisman E. Mutagive formation in deep fat fried foods as a function of frying conditions. JAOCS. 1983;60:576–80.

[105] Rossel JB. Vegetable oils and fats. 1st ed. London: C.M.E. Casterberg; 1984. p. 263–65.

[106] Chan HWS, Cotton DT. The mechanism of autoxidation. Orland: Academic Press, Inc; 1987. p. 49.

[107] Ruiz MC, Margaret TM, Dobarganea MC. Quantitative and distribution of altered fatty acids in frying fats. J Am Oil Chem Soc. 1995;72(10):1171–76.

[108] Muhammad N, Bamishaiye E, Bamishaiye O, Usman L, Salawu M, Nafiu M, et al. Physicochemical properties and fatty acid composition of Cyperus esculentus (Tiger Nut) Tuber Oil. Biores Bull. 2011;5:51–4.

[109] Lea CH. The oxidative deterioration of food lipids. In: Schultz HW, Day EA, Sinnhuber RO, editors. Symposium on food: lipids and their oxidation. Westport, Connecticut: The AVI Publishing Company, Inc; 1962. p. 3–28.

[110] Olaposi AR, Adunni AO. Nutritional and physico-chemical properties of Bombax glabrum Seeds. Pak J Nutr. 2010;9(9):856–57.

[111] Aluyor EO, Aluyor P, Ozigagu CE. Effect of refining on the quality and composition of groundnut oil. Afr J Food Sci. 2009;3(8):201–5.

[112] Kirk RS, Sawyer R. Pearson’s composition and analysis of foods. 9th edn. England: Addison Wesley Longman Ltd; 1991. p. 9–29, 608–40.

[113] Radwan SMA, Awad NM. Effect of soil Amendment with various organic wastes with multi-biofertilizer on yield of peanut plants in sandy soil. J Agric Sci Mansoura Univ. 2002;27(5):3129–38.

[114] Safwat MSA, Badran FS. The efficiency of organic and bio-fertilizers, in comparison with chemical fertilization, on growth, yield, and essential oil of cumin plants. The10th Conference Medicinal and Aromatic Plants in Arab Countries, Sustainable Development; 14–16 December, 2002. p. 11–9.

[115] Mekki BB, Amal G, Ahmed G. Growth, yield, and seed quality of soybean (Glycine max L.) as affected by organic, biofertilizer, and yeast application. Res J Agric Biol Sci. 2005;1(4):320–4.

[116] Azzaz NA, Hassan EA, Hamad EH. The chemical constituent and vegetative and yielding characteristics of fennel plants treated with organic and bio-fertilizer instead of mineral fertilizer. Aust J Basic Appl Sci. 2009;3(2):579–87.

[117] Brady NC, Weil RR. The nature and properties of soil. 14th edn. New Jersey: Prentice Hall; 2008.

[118] Parikh SJ, James BR. Soil: The foundation of agriculture. Nat Educ Knowl. 2012;3(10):2.

[119] Son HJ, Park GT, Cha M, Hea MS. Solubilization of insoluble inorganic P by a novel salt and pH tolerant Pantoea agglomerans R- 42 Isolated from soybean rhizosphere. Biores Technol. 2006;97:204–10. 10.1016/j.biortech.2005.02.021 16171676

[120] Lakshmi CS, Sreelatha T, Rani T, Rao SRK, Naidu NV. Effect of organic manures on soil fertility and productivity of sugarcane in North Coastal Zone of Andhra Pradesh. Indian J Anim Res. 2011;45:307–13.

[121] Arnold SL, Doran JW, Schepers J, Wienhold B, Ginting D, Amos B, et al. Portable probes to measure electrical conductivity and soil quality in the field. Commun Soil Sci Plant Anal. 2005;36:2271–87.

[122] Smith JL, Doran JW. Measurement and use of pH and electrical conductivity for soil quality analysis. In: Doran JW, Jones AJ, editors. Methods for assessing soil quality. Vol. 49, SSSA, Madison: Soil Science Society of America Journal; 1996. p. 169–85.

[123] Mandali R, Subbaiah V. Available nutrient status in rice growing soils of various Mandals in Krishna District. Int J Chem Stud. 2020;8(4):2745–8.

[124] Sushila A, Yadav BL, Bhuli DG, Jitendra SB. Effect of soil salinity, P, and biofertilizers on physical properties of soil, yield attributes and yield of cowpea Vigna unguiculata (L.) Wilczek. J Pharmacogn Phytochem. 2017;6(4):169–1695.

[125] Shaban Kh A, Attia AM. Evaluation of bio- and chemical fertilizers applied to corn grown on a saline sandy soil. Minufiya J Agric Res. 2009;34(3):1311–26.

[126] Carter MR, Gregorich EG, (Eds.). Soil sampling and methods of analysis. Boca Raton, FL, USA: CRC Press, Inc; 2007. p. 607–35.

[127] Carter MR, Stewart BA, (Eds). Structure and Organic Matter Storage in Agricultural Soils (Advances in Soil Science). 1st edn. Boca Raton: CRC Press; 1995. p. 15–41.

[128] Musinguzi P, Ebanyat P, Tenywa JS, Basamba TA, Tenywa MM, Mubiru DN. Critical soil organic carbon range for optimal crop response to mineral fertiliser nitrogen on a ferralsol. Expe Agric. 2016;52(4):635–53.

[129] Kohli I, Mohapatra S, Kumar P, Goel A, Varma A, Joshi NC. Role of bacterial endophytes in the promotion of plant growth. In: Singh AK, Tripathi V, Shukla AK, Kumar P, editors. Bacterial endophytes for sustainable agriculture and environmental management. Singapore: Springer; 2022.

[130] Mengel K, Kirkby EA, Kosegarten H, Appel T. Principles of plant nutrition. Dordrecht: Kluwer Academic Publishing Co., Inc. Redwood City, CA; 2001. p. 100–19.

[131] Mahapatra DM, Satapathy KC, Panda B. Biofertilizers and nanofertilizers for sustainable agriculture: Phycoprospects and challenges. Sci Total Env. 2022;803:149990. 10.1016/j.scitotenv.2021.149990 34492488

[132] Giller KE, Hijbeek R, Andersson JA, Sumberg J. Regenerative agriculture: an agronomic perspective. Outlook Agric. 2021;50:13–25. 10.1177/0030727021998063 PMC8023280 33867585

[133] Ayaga G, Todd A, Brookes PC. Enhanced biological cycling of P increases its availability to crops in low-input sub-saharan farming systems. Soil Biol Biochem. 2006;38:81–90.

